# Probing soft X-ray induced photoreduction of a model Mn-complex at cryogenic conditions

**DOI:** 10.1107/S1600577524012189

**Published:** 2025-02-03

**Authors:** Kuntal Chatterjee, Sang-Jun Lee, Li-Cheng Kao, Margaret D. Doyle, Charles J. Titus, Stephen R. Leone, Junko Yano, Vittal K. Yachandra, Philippe Wernet, Jan F. Kern

**Affiliations:** a Molecular Biophysics and Integrated Bioimaging Division Lawrence Berkeley National Laboratory Berkeley CA94720 USA; b Department of Chemistry University of California Berkeley Berkeley CA94720 USA; c Chemical Sciences Division Lawrence Berkeley National Laboratory Berkeley CA94720 USA; d SLAC National Accelerator Laboratory Menlo Park CA94025 USA; e National Institutes of Standards and Technology Gaithersburg MD20899 USA; f Department of Physics University of California Berkeley Berkeley CA94720 USA; g Department of Physics and Astronomy Uppsala University 75120Uppsala Sweden; University of Essex, United Kingdom

**Keywords:** *L*-edge spectroscopy, transition metals, radiation damage

## Abstract

Soft X-ray absorption spectroscopy of first row transition elements at their respective *L*-edges provides important information about the oxidation and spin states of the metal centers. However, the associated sample damage in radiation-sensitive samples substantially alters the electronic and chemical structures of redox-active metal centers. Here, we measure the soft X-ray spectrum of the model Mn^III^(acac)_3_ complex containing a redox-active Mn^III^ metal center in an octahedral environment with a superconducting transition-edge sensor detector. To reduce the secondary damage resulting primarily from the diffusion of radicals and electrons, the spectra are collected at 30 K and 80 K on solid samples. Starting from the first scan, we detect the contribution of X-ray induced sample damage leading to a change in the Mn^II^ intensity. However, at low temperatures, particularly at 30 K, we do not observe a gradual increase in the radiation damage with successive scans with the X-ray beam at the same spot. At our estimated dose of 90 kGy, we find 62% of Mn^III^(acac)_3_ is still intact at 30 K. However, at room temperature, we see a gradual increase in radiation damage with increasing numbers of scans at the same spot, which is consistent with the possibility of increased diffusion rates of secondary radicals and electrons as noted in other studies.

## Abstract 2

This controlled radiation damage study at different temperatures allows the approaches to measuring radiation-damage-free/limited spectra from redox-sensitive metalloenzymes and metal complexes in the soft X-ray regime at synchrotrons to be refined.

## 1. Introduction

The high brilliance of third-generation synchrotron and X-ray free-electron lasers (XFEL) facilitates new types of experiments to gain insights into structural and functional information of biomolecules (Bilderback *et al.*, 2005 ▸; Young *et al.*, 2010 ▸; Coppens, 2011 ▸; Zimmermann *et al.*, 2020 ▸; Bergmann *et al.*, 2021 ▸). However, the associated increase in X-ray flux can also lead to X-ray induced sample damage (XISD) to biomolecules (Garman & Weik, 2019 ▸; Henderson, 1995 ▸; Lomb *et al.*, 2011 ▸) and other redox-sensitive metal samples. This in turn changes the electronic and geometric structures of the probed species (Yano *et al.*, 2005 ▸; Pushkar *et al.*, 2008 ▸; Hersleth & Andersson, 2011 ▸). Therefore, it is crucial to consider how much radiation a sample can withstand before being damaged beyond an acceptable limit. One of the measures to assess XISD in X-ray crystallography has been the loss of intensity and resolution of the diffraction signal. XISD is often correlated with the amount of X-ray dose accumulated by the sample after X-ray irradiation. This dose is defined as the ratio of absorbed photon energy per sample mass and is measured in units of Gray (Gy, where 1 Gy = 1 J kg^−1^). In X-ray protein crystallography, an X-ray dose of 20 MGy (2 × 10^7^ Gy) is reported where 50% of the initial sample is damaged (the so-called *D*_0.5_ value) causing the diffraction power to drop by half (Henderson, 1990 ▸). This limit is known as the Henderson limit. Later, this limit was changed to 30 MGy (Owen *et al.*, 2006 ▸).

After the interaction of X-ray photons with matter, a photoelectron is released and leaves behind a core-hole, which, besides fluorescence decay, undergoes Auger decay through the emission of another electron in the femtosecond time scale (Sankari *et al.*, 2020 ▸; Jahnke *et al.*, 2021 ▸; Siegbahn *et al.*, 1975 ▸; Piancastelli *et al.*, 1999 ▸). Past studies on radiolysis of water in the gas phase revealed that a water di-cation (H_2_O^2+^) resulting from initial ionization by X-rays undergoes fragmentation *via* two different channels within tens of femtoseconds,
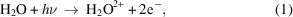

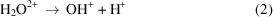

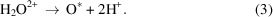
The first channel [equation (2)] is known as two-body fragmentation and the second [equation (3)] is called three-body fragmentation (Malegat *et al.*, 1997 ▸; Akoury *et al.*, 2007 ▸; Reedy *et al.*, 2018 ▸). Interestingly, in the liquid environment, an additional channel occurs where the electrons resulting from the initial process [equation (1)] ionize the surrounding solvent leading to valence ionized water, H_2_O^+^, which rapidly dissociates (with a time constant of 50 fs) into a hydro­nium ion and a hydroxyl radical (Kamarchik *et al.*, 2010 ▸; Marsalek *et al.*, 2011 ▸; Loh *et al.*, 2020 ▸),
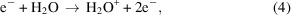

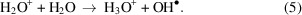
The secondary electrons and radicals subsequently undergo radiolytic reactions typically known as secondary damage. This secondary damage includes ruptures of chemical bonds, reductions of redox-active metal centers in proteins, and irreversible changes in local molecular structures, which all may cause long-range structural rearrangement in protein crystals that ultimately reduce diffraction intensity (Weik *et al.*, 2000 ▸; O’Neill *et al.*, 2002 ▸; Garman, 2010 ▸; Sutton *et al.*, 2013 ▸).

The secondary damage caused by electrons and radicals is thought to be mostly diffusion controlled (O’Neill *et al.*, 2002 ▸; Garman, 2003 ▸). Therefore, a reduction in temperature causes a substantial reduction of radiation damage. However, the high brilliance of third-generation synchrotron and XFEL sources still causes visible XISD to samples even at 100 K motivating the necessity to collect data at even lower temperature in the range 10–20 K (Garman, 1999 ▸). We further note that secondary damage cannot be eliminated completely even at 10 K as seen from a past study of the photosystem II (PS II) protein complex (Yano *et al.*, 2005 ▸). In such cases, the introduction of the concept of ‘probing before destroying’ at XFELs helps to avoid XISD (Chapman *et al.*, 2014 ▸, 2011 ▸; Boutet *et al.*, 2012 ▸; Kern *et al.*, 2013 ▸; Alonso-Mori *et al.*, 2012 ▸; Mitzner *et al.*, 2013 ▸; Kubin *et al.*, 2017 ▸). In this approach, probing the sample with fs X-ray pulses and replacing the entire sample between successive XFEL laser pulses rules out the possibility of XISD (possible non-linear effects of the short and intense XFEL pulses need to be avoided as well). In this way, damage-free data are measured even at room temperature. At synchrotrons, XISD is always a limiting factor due to the long (ps) X-ray pulses and high repetition rates.

It is noteworthy to mention that, in contrast to the radiation-dependent decrease in X-ray diffraction signal due to loss of long-range structural order, local chemical changes, such as alteration of oxidation states and bond lengths, already start to occur at lower X-ray doses (Yano *et al.*, 2005 ▸; Corbett *et al.*, 2007 ▸; George *et al.*, 2012 ▸; van Schooneveld & DeBeer, 2015 ▸). A previous high-resolution X-ray diffraction study reported a *D*_0.5_ value of 0.2–0.4 MGy at cryogenic temperatures, which is two orders of magnitude lower than the Henderson limit (30 MGy) (Sutton *et al.*, 2013 ▸). A number of studies involving hard X-ray absorption spectroscopy (XAS) and X-ray emission spectroscopy (XES) have reported X-ray induced reduction of high-valent metal centers with *D*_0.5_ limits ranging between 0.1 and 10 MGy with lower *D*_0.5_ values at room temperature and higher dose-limits at cryogenic temperatures (Yano *et al.*, 2005 ▸; Davis *et al.*, 2012 ▸). Interestingly, there are a limited number of studies on XISD in the soft X-ray range (0.1–1 keV). A previous XAS study in the soft X-ray range on various iron complexes in the solid phase reported a relatively large range for the *D*_0.5_ value, between 49 and 4200 MGy (van Schooneveld & DeBeer, 2015 ▸; George *et al.*, 2008 ▸). Further XAS studies using synchrotron soft X-ray pulses on a manganese compound, Mn^III^(acac)_3_ {manganese(3+) tris-[(2*Z*)-4-oxo-2-penten-2-olate]} in the solution phase with a liquid jet, where the sample is continuously being replaced, reported a damage-free partial fluorescence yield detected XAS (PFY-XAS) and transmission detected Mn^III^ spectrum at a dose limit of 5 kGy at room temperature (Kubin, Guo, Kroll *et al.*, 2018 ▸; Kubin, Guo, Ekimova *et al.*, 2018 ▸). With a non-flowing liquid sample in a cell, the same group reported an XAS study that showed 10% photoreduced Mn^III^ signal with a dose around 30 kGy at room temperature (Kubin, Kern, Guo *et al.*, 2018 ▸).

Herein, we report an XAS study carried out in the soft X-ray energy range to understand the photochemical changes after X-ray interaction with Mn^III^(acac)_3_ samples using synchrotron soft X-ray pulses at sample temperatures of 80 K and 30 K. Unlike previous experiments with this model complex, which were conducted in the solution phase, we performed this study using solid powder samples to investigate whether cooling the solid sample to cryogenic temperatures would help avoid XISD. We expected that charge migration may be limited to only tunneling effects in the cold solid phase (O’Neill *et al.*, 2002 ▸; Kubin, Kern, Guo *et al.*, 2018 ▸). Therefore, the diffusion rate of electrons and radicals should be smaller compared with the liquid phase experiments leading to reduced XISD. Another possibility could be the reduced diffusion of chain reaction propagation, which is well known from gas phase reactions (Miller & Klippenstein, 2003 ▸), that minimizes radiation damage at cryogenic temperature. Finally, unlike the liquid jet experiments, samples are not replaced continuously; rather, they were raster scanned to minimize the X-ray dose. It is important to note that the stability of these jets is limited over time, and sample consumption is relatively high. In our current study, we propose an alternative approach that aims to mitigate radiation damage by utilizing cryogenic temperatures. Furthermore, rather than employing a large quantity of the sample, we can create a solid film, which allows us to obtain the necessary signal while significantly reducing sample consumption. Although our measured spectral range is between 636 eV and 659 eV, which includes 2*p*–3*d* transitions at the Mn *L*-edge (*L*_3_ and *L*_2_), most of the scans were carried out between 636 eV and 646 eV. In this shorter energy limit we sensitively probe X-ray induced reduction of Mn^III^ to Mn^II^, where the latter has a characteristic *L*_3_-edge absorption at 639.6 eV, which is 2 eV lower than the Mn^III^ absorption, which is located at 641.6 eV (Kubin, Guo, Kroll *et al.*, 2018 ▸). We note that different possible X-ray induced effects could occur within the metal-containing complexes. To name a few, photoreduction, photooxidation, mass loss, bond breaking and so on (van Schooneveld & DeBeer, 2015 ▸). Various factors control these processes, for instance metal oxidation state, temperature, pressure and so on. Multiple previous studies reported that high-valent metal centers are prone to be photoreduced after interaction with X-ray pulses (van Schooneveld & DeBeer, 2015 ▸; George *et al.*, 2008 ▸; Kubin, Kern, Guo *et al.*, 2018 ▸). In our measured spectral range, in addition to Mn, light atoms (H, C, O) in the sample absorb X-ray energy leading to the generation of primary and secondary electrons that cause Mn^III^ → Mn^II^ photoreduction (Kubin, Kern, Guo *et al.*, 2018 ▸). Probing the ligand atom *K*-edges (O, C) could provide additional details to the XISD mechanism, which is however beyond the scope of this study. We further acknowledge that the radiation damage leads to a rather inconsistent data set resulting from an uncontrollable radical/electron-induced process(es). Therefore, it is beyond our scope to discuss the actual underlying X-ray induced damage mechanism at present. Keeping this in mind, we strongly believe that every radiation damage related study is important to address the underlying limitations associated with X-ray–matter interactions.

Herein, we primarily focus on the metal *L*-edge to estimate whether an XISD occurs which is consistent with past studies and eventually report the proportion of damage-free and photo-reduced metal centers (van Schooneveld & DeBeer, 2015 ▸; George *et al.*, 2008 ▸; Kubin, Kern, Guo *et al.*, 2018 ▸). This work on controlled illumination of Mn^III^ samples demonstrates soft X-ray radiation induced reduction of Mn^III^ in the solid phase at cryotemperatures. The findings are crucial for designing ideal experimental conditions to record damage-free *L*-edge spectra of high-valent Mn-containing transition-metal complexes and metalloproteins (such as PS II) in the solid phase (or frozen metalloenzymes) using soft X-rays from synchrotron and XFEL sources.

## 2. Experimental methods

### 2.1. Sample preparation

Mn^III^(acac)_3_ {manganese(3+) tris-[(2*Z*)-4-oxo-2-penten-2-olate]} [acetyl­acetonate ligands abbreviated as (acac)^−^] as crystalline powder was purchased from Fischer Scientific (purity 97%) and used without further purification. We also obtained powder of Mn^II^(acac)_2_ {manganese(2+) bis-[(2*Z*)-4-oxo-2-penten-2-olate]} as a reference sample to estimate the radiation-induced reduction of Mn^III^(acac)_3_. We held these powder samples on sticky carbon tapes that were attached to a sample holder made of aluminium. Then we loaded the sample holder into the load-lock chamber and pumped it down to ∼1 × 10^−7^ Torr.

### 2.2. Experimental setup

Mn *L*-edge XAS spectra were collected at beamline 10-1 of the Stanford Synchrotron Radiation Lightsource (SSRL). The sample holder sitting in the load-lock chamber was then transferred to the main chamber (∼5 × 10^−9^ Torr) and gradually cooled down to 80 K using a liquid-nitrogen-cooled cryostat for one series of measurements or to 30 K using a liquid-helium-cooled cryostat for another series of measurements. The spatial coordinates of the sample holder with respect to the incident X-ray beam were determined using the beam and a photodiode located downstream of the beam. Once this procedure was completed, samples were placed at the sample–beam interaction point with a desired angle of incidence. In the present work, the samples were positioned at an angle of 45° with respect to the beam. The X-ray beam’s polarization was horizontal, which is consistent with the polarization direction in the past PFY-XAS liquid jet experiments (Kubin, Guo, Kroll *et al.*, 2018 ▸). The detector was positioned in the horizontal plane at a 90° angle relative to the incident beam direction, and the incident angle of the beam with respect to the sample surface was 45°. The XES energy range for the 2*p*3*d* PFY-XAS was 600–665 eV. The reference spectra are reported to have been obtained by integrating Mn-*L*_α,β_. Therefore, our XES energy range should essentially match with the past PFY-XAS liquid jet experiments, as there is no other signal that could be unintentionally included (Kubin, Guo, Kroll *et al.*, 2018 ▸). The incident beam was monochromated by a 1000 lines mm^−1^ ruling spherical grating monochromator (SGM), providing an estimated energy resolution (Δ*E*_beam_) in the range 0.25–0.40 eV.

The energy of the X-ray beam was calibrated by setting the energy of the Mn^II^ peak from the beamline reference at 639.6 eV (Kubin, Guo, Kroll *et al.*, 2018 ▸). The beamline reference consisted of a thin film of Mn compounds containing transition metals deposited on a silicon wafer. Positioned upstream, this reference material partially blocked the incident beam flux (approximately 2% of the total flux and it was kept as it is during the sample measurement), enabling the simultaneous acquisition of the TEY-XAS signal from the reference for energy calibration.

XAS measurements were conducted in two modes: total electron yield (TEY) and PFY (Lee *et al.*, 2019 ▸). Both TEY and PFY modes were utilized at room temperature. After cooling the samples, we collected the data in PFY mode as the TEY signal became unavailable due to unintended interference between the current amplifier and the temperature controller used. This is why we restrict ourselves to discussing the results obtained through PFY mode.

A transition-edge sensor (TES) spectrometer was operated in the so-called maximum efficiency mode, utilizing approximately 190 channels to achieve high sensitivity. A relatively large solid angle, excellent energy sensitivity, and dark-count-free nature make the TES detector ideal to acquire XAS, resonant inelastic X-ray scattering (RIXS) and XES signals from dilute and/or radiation-damage-sensitive samples (Doriese *et al.*, 2017 ▸; Uhlig *et al.*, 2015 ▸; Qiao *et al.*, 2017 ▸; Bufon *et al.*, 2018 ▸). In this work, the energy resolution of the TES spectrometer was estimated to be approximately 2.5 eV FWHM (full width at half-maximum) at 750 eV. The TES spectrometer was energy calibrated approximately every 8 h using fluorescence lines from a TES calibration reference, consisting of light element compounds and transition-metal oxides excited by the 750 eV X-ray beam. Further details of the TES spectrometer can be found elsewhere (Lee *et al.*, 2019 ▸).

XAS measurements mainly focused on scanning the incident energy around the *L*_3_-edge of Mn. The scan range covered 7.5 eV with energy increments of 0.5 eV in off-peak regions and 0.2 eV in on-peak regions to ensure high-resolution spectra while minimizing radiation dose on the sample. Data were collected for 0.5 s at each energy point and the samples were raster scanned during data acquisition with the aim to further minimize sample damage during measurements. For this, the sample holder was moved horizontally to an unexposed neighboring spot after completing XAS measurements at one spot. We spent <30 s on each spot. After measuring approximately eight spots in a row, the sample holder moved vertically to a new row, forming a zigzag pattern. We raster scanned over roughly 100 spots on average to record one spectrum at low temperatures. The horizontal and vertical gaps between spots were set at 2 mm, which was significantly larger than the size of the incident beam (∼1 mm horizontally and ∼0.5 mm vertically). To determine the dose at which noticeable changes in spectral shape occurred, each spot was measured consecutively at least three times.

Different entrance and exit slit openings ranging from 30 µm to 60 µm were employed. For the presented spectra, the lowest dose setting with a 30 µm opening was used, while a higher dose setting with a 60 µm opening was used for some measurements of intentional sample damage.

Two different flux settings were employed in the present study. The high photon flux setting corresponds to 5 × 10^10^ photons s^−1^ while the low photon flux setting corresponds to 3 × 10^9^ photons s^−1^, which was achieved by a small slit opening and intentional detuning of one of the upstream X-ray optics. The energy step size at 80 K and high flux settings are smaller (0.1 eV) across the scan range compared with those collected at 30 K and low flux settings (0.2 eV in the on-peak regions) discussed earlier.

## 3. Results and discussion

**Figure 1:**
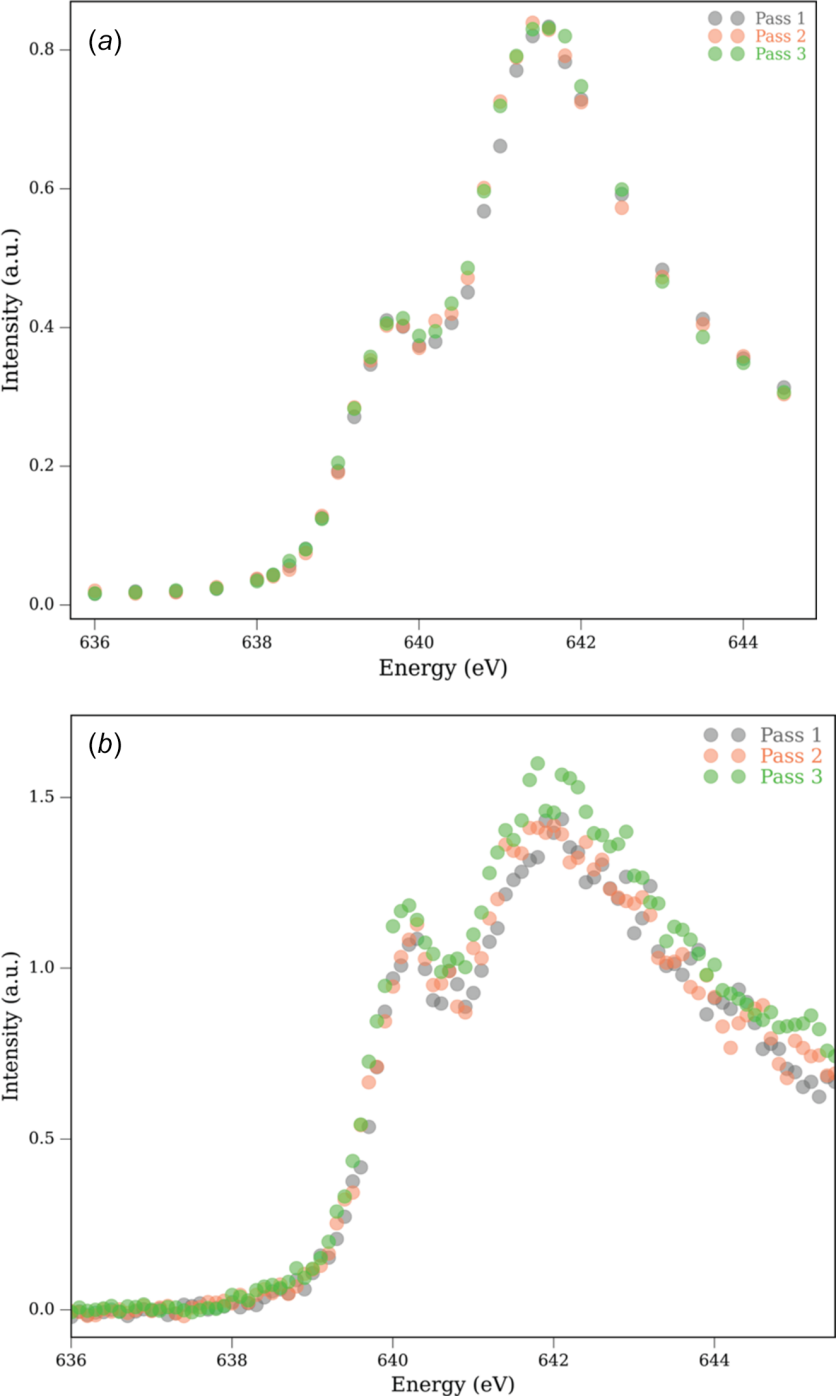
Mn *L*_3_-edge PFY-XAS spectra of Mn^III^(acac)_3_ measured at (*a*) 30 K and (*b*) 80 K with low photon flux setting. The figure legends correspond to the scan (pass) number at a particular spot.

Figs. 1 ▸(*a*) and 1(*b*) show PFY-XAS data of Mn^III^(acac)_3_ collected at 30 K and 80 K, respectively, in the Mn *L*_3_-edge region. The Mn *L*-edge signal corresponds to the 2*p*^6^3*d*^*n*^ → 2*p*^5^3*d*^*n*+1^ core-to-valence absorption resonances, where an Mn 2*p* electron is excited to the 3*d*-derived molecular orbitals. This *L*-edge signal is split by roughly 10 eV due to spin–orbit coupling leading to *L*_3_- and *L*_2_-edge transitions. In Mn^III^(acac)_3_, the *L*_3_-edge maximum appears at 641.6 eV, while it is at 639.6 eV in the Mn^II^ complex. This is consistent with the previously reported PFY-XAS *L*-edge spectra of damage-free Mn^III^(acac)_3_ and Mn^II^(acac)_2_ collected with a liquid jet sample delivery approach (Kubin, Guo, Kroll *et al.*, 2018 ▸; Kubin, Guo, Ekimova *et al.*, 2018 ▸). As the *L*_3_ signals of Mn^III^(acac)_3_ and Mn^II^(acac)_2_ are 2 eV apart, they act as marker bands for Mn^III^ and Mn^II^ sites. For both complexes, there is a broad *L*_2_ signal around 650 eV with less pronounced difference between Mn^III^ and Mn^II^ (Kubin, Guo, Kroll *et al.*, 2018 ▸). Therefore, we mainly focus on the *L*_3_-edge region between 636 eV and 646 eV to follow XISD.

The reported *L*_3_ XAS spectrum of Mn^III^(acac)_3_ acquired in the transmission mode shows a prominent pre-edge feature at 639.6 eV. This signal overlaps with an XAS feature of Mn^II^(acac)_3_ measured in transmission mode (Kubin, Guo, Ekimova *et al.*, 2018 ▸). Interestingly, this pre-edge signal of Mn^III^(acac)_3_ is substantially reduced in the damage-free XAS spectrum recorded in PFY mode (Kubin, Guo, Kroll *et al.*, 2018 ▸). However, there is a weak but non-zero pre-edge signal of Mn^III^(acac)_3_ at 639.6 eV. Previous studies demonstrated such a change in intensity based on the signal recorded either in PFY or transmission mode. We note that such a change in the PFY-XAS mode signal compared with the transmission-mode arises from the state-dependent fluorescence decay channels, causing relative peak-intensities deviating from the absorption cross-sections measured in the transmission mode (De Groot *et al.*, 1994 ▸; Wernet *et al.*, 2012 ▸; Achkar *et al.*, 2011 ▸; Kurian *et al.*, 2012 ▸). The state-dependent fluorescence decay variation could be 400% stronger compared with the actual transition cross sections (*i.e.* the XAS spectrum measured in the transmission mode) (De Groot *et al.*, 1994 ▸). Therefore, in our present work, we primarily assign any strong signal of the Mn^III^(acac)_3_ complex at 639.6 eV as resulting from photo-reduction of Mn^III^ → Mn^II^ (XISD) (Kubin, Guo, Kroll *et al.*, 2018 ▸; Kubin, Guo, Ekimova *et al.*, 2018 ▸; Kubin, Kern, Guo *et al.*, 2018 ▸). However, we do not exclude other explanations of the pre-edge signal being part of undamaged Mn^III^ species together with the contribution of photo-reduced Mn^III^, especially given that the simulated spectrum in the reference paper also shows a pre-edge feature (Kubin, Guo, Kroll *et al.*, 2018 ▸). However, the presence of a published Mn^III^(acac)_3_ spectrum with a much reduced pre-edge signal has made us use this spectrum as a baseline for our analysis and led us to adopt the explanation where the Mn^III^(acac)_3_ sample is photo-reduced. Finally, we checked the sample purity by measuring the *K*-edge XANES spectra of our Mn^III^(acac)_3_ and Mn^II^(acac)_2_ samples. Both spectra are completely different. We did not observe any impurity originating from Mn^II^ contamination in our Mn^III^(acac)_3_ sample and vice versa. This established that the sample used for the *L*-edge measurement reported here contained only Mn in the oxidation state 3+, as expected (see Fig. S1 of the supporting information). Based on the previous X-ray induced high-valent metal reduction model (Kubin, Kern, Guo *et al.*, 2018 ▸), we primarily consider the signal at 639.6 eV resulting from Mn^II^(acac)_2_ and use this to estimate the radiation damage of pure Mn^III^(acac)_3_ which has a maximum signal at 641.6 eV.

**Figure 2:**
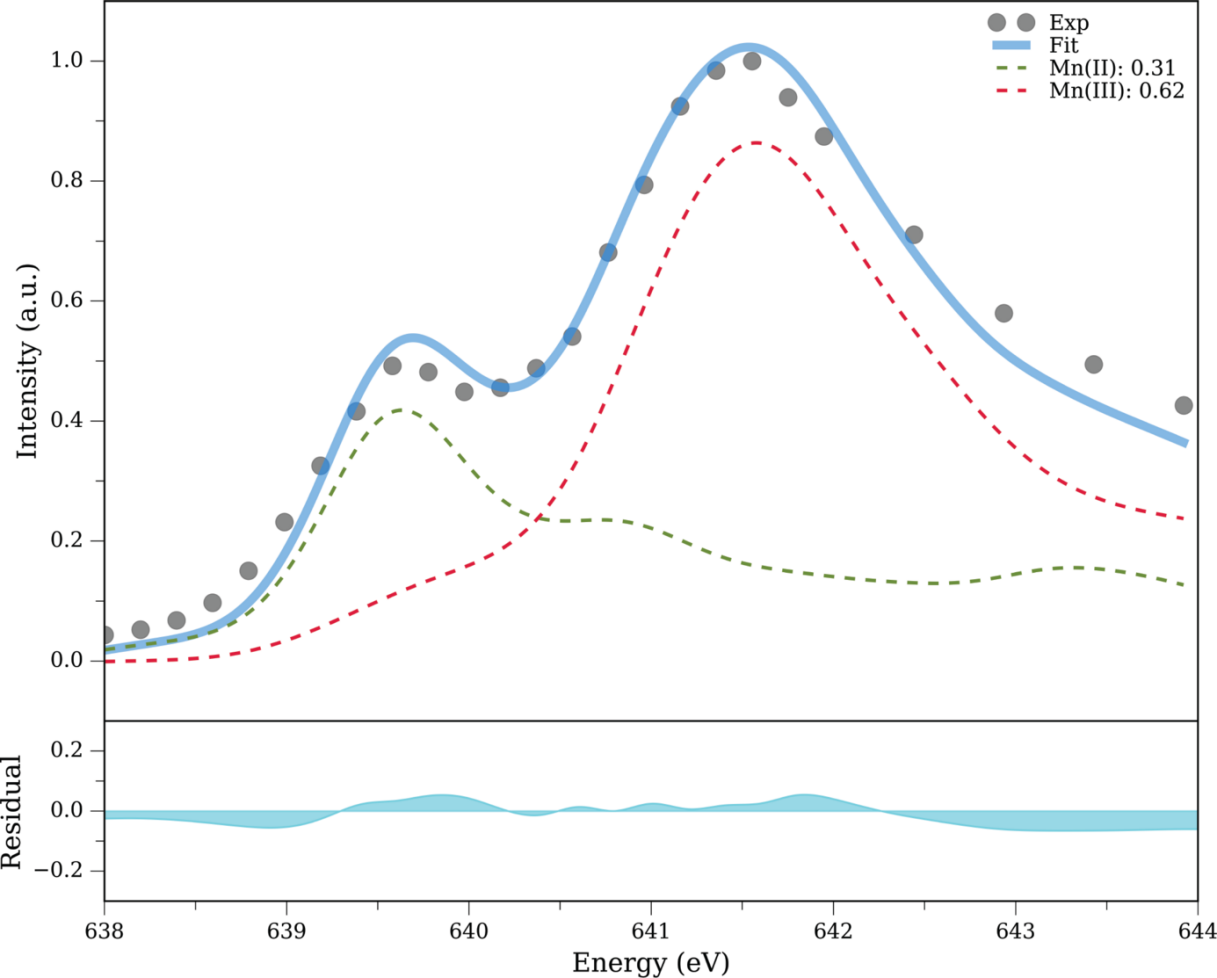
Fit of the normalized Mn *L*-edge PFY-XAS spectrum of Mn^III^(acac)_3_ (circles) complex measured at 30 K with linear combinations of damage-free PFY-XAS spectra of Mn^II^(acac)_2_ (green) and Mn^III^(acac)_3_ (red) taken from Kubin, Guo, Kroll *et al.* (2018 ▸). Fit residuals are shown in the bottom panel.

The first scan (pass 1) recorded with low photon flux setting in the PFY-XAS spectrum of Mn^III^(acac)_3_ recorded at 30 K on a particular spot shows a visible signal at 639.6 eV along with the strongest intensity at 641.6 eV, which is the characteristic *L*_3_-edge maximum of Mn^III^ [Fig. 1 ▸(*a*)]. The feature at the lower energy corresponds to a contribution from Mn^II^, which primarily arises from radiation-induced reduction of the Mn^III^ center. Fig. 2 ▸ demonstrates how we quantify the amount of reduced Mn^II^ species in the sample by fitting undamaged Mn^III^ and Mn^II^ spectra to our measured spectra. Further details of our fitting approach are explained in Section S1 of the supporting information. We estimate 31% of Mn^II^ and 62% of Mn^III^ to be present after the first PFY-XAS scan in the low-flux setting at 30 K (the remaining 7% can be attributed to unwanted minor impurities that might be present in this damaged sample; see the supporting information). The fit is in good agreement with the experimental spectrum (see the small residuals of below 10% in the bottom panel of Fig. 2 ▸). Interestingly, when we measured the spectrum at the same spot with low flux settings for the second and third scans (passes), we did not observe any increase in the Mn^II^ signal [Fig. 1 ▸(*a*)] in our present signal level (we can clearly observe changes above 5%). In other words, we see an initial damage during the first scan that seems to saturate and not increase further with subsequent scans. We tentatively argue that this scenario could arise from a reduced diffusion rate of radicals and electrons produced after the interaction between soft X-ray pulse and sample at this low temperature (O’Neill *et al.*, 2002 ▸; Kubin, Kern, Guo *et al.*, 2018 ▸). Alternatively, the sample damage could occur from a chain propagation reaction, and this is well known to be temperature dependent (Miller & Klippenstein, 2003 ▸). Both these possibilities demonstrate decreased mobility of the initially produced electrons and radicals at cryogenic temperatures. As a result, the signal arising from the primary radiation damage occurs, changes the spectrum, and saturates, while secondary radiation damage initially already saturates with no further increase, or does not even occur on a significant level. However, this remains an open question at this point.

A similar situation is also seen when we measure the spectra at 80 K with low photon flux setting. Here, the Mn^II^ contribution is estimated to be around 42% [Mn^III^ 51%; Fig. 1 ▸(*b*)]. With increasing number of scans, we see a slight increase in the Mn^II^ contribution, which however lies within the experimental and/or fit error (5.1%). The slight increase in Mn^II^ signal (∼11%) at 80 K compared with the 30 K data reflects stronger XISD and is attributed to a higher mobility of ions and electrons at elevated temperatures.

**Figure 3:**
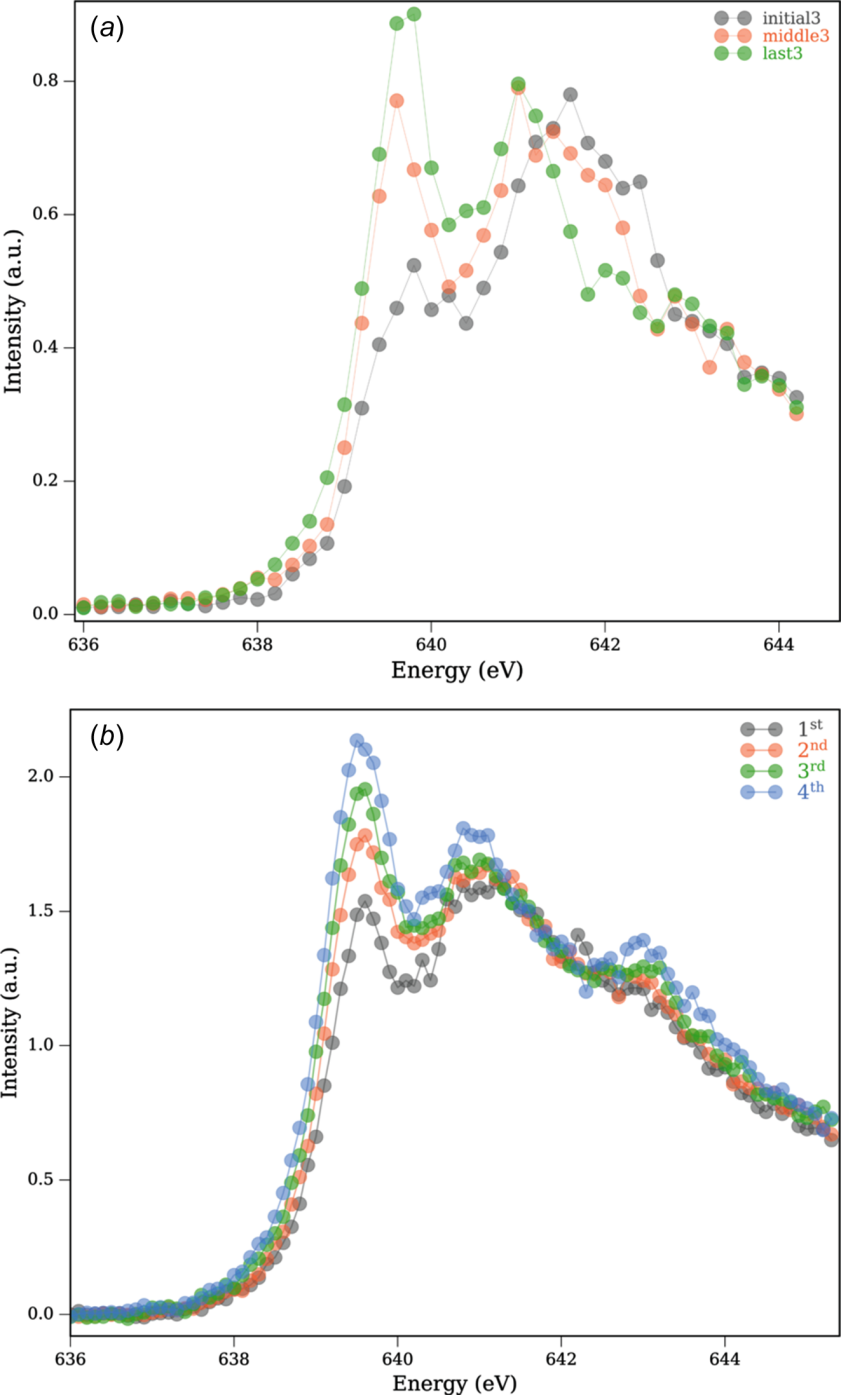
Mn *L*_3_-edge PFY-XAS spectra of Mn^III^(acac)_3_ measured at room temperature with (*a*) low flux and (*b*) high flux settings. The figure legends correspond to the scan number at a particular spot. In (*a*), out of the total of 50 scans taken at the same spot, averages of the initial three, middle three and last three scans (passes) are shown at the same sample position. In (*b*), only four scans (passes) are recorded at the same sample position.

**Figure 4:**
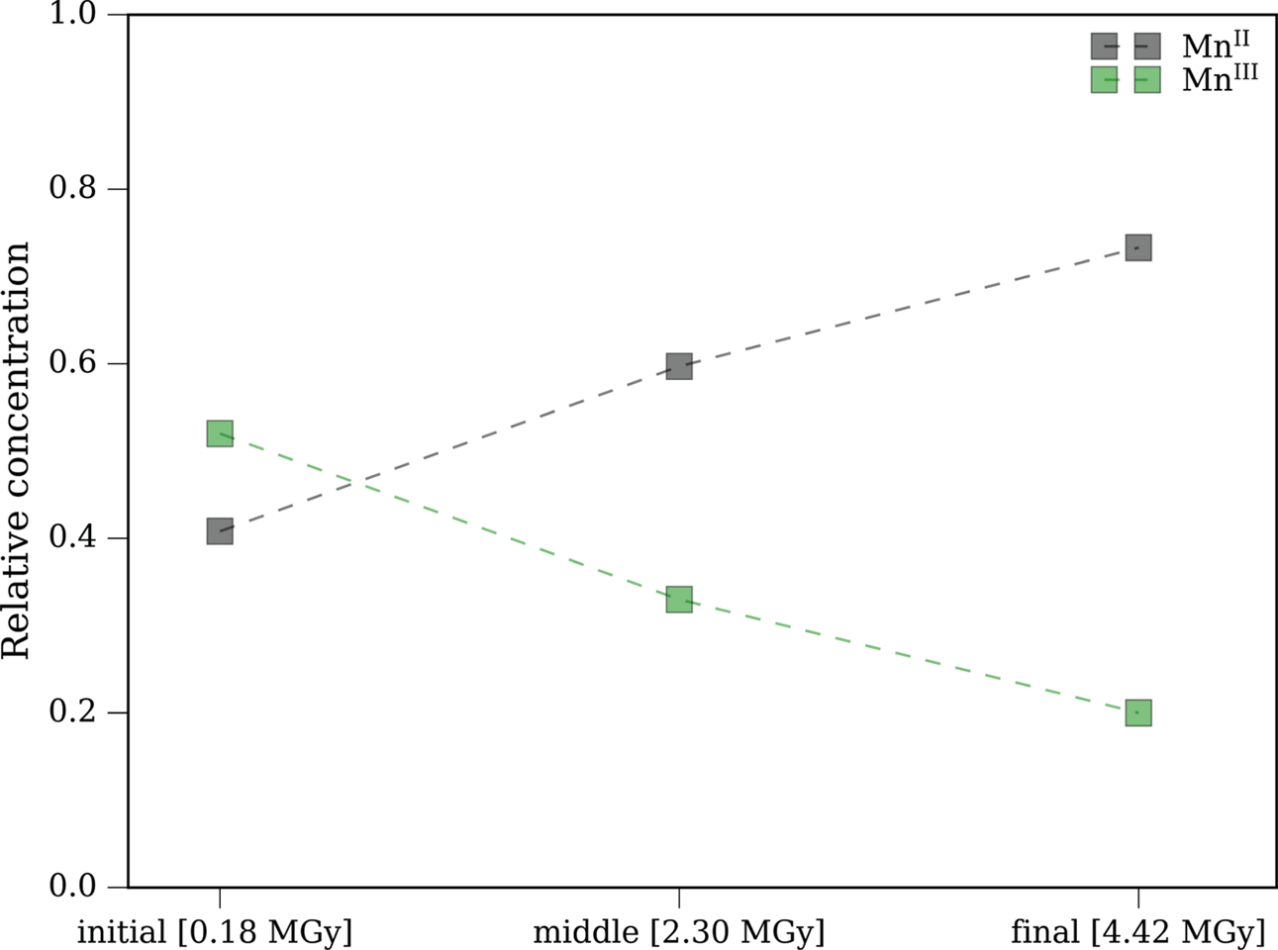
Gradual change in relative concentration of Mn^III^ and Mn^II^ species with increasing number of scans at the same spot (fitting uncertainty 4–6.5%). The spectra are measured at room temperature with low photon flux setting. Total accumulated doses are reported in square brackets.

When we measure at room temperature, a different situation is encountered. From Figs. 3 ▸(*a*) and 3(*b*) we see a gradual increase in the Mn^II^ signal with increasing number of scans at the same spot. Data in Fig. 3 ▸(*a*) were recorded at the low photon flux setting, while data in Fig. 3 ▸(*b*) were recorded with high photon flux (50% flux of this setting) setting. We fit the data using the same procedure (Section S1 of the supporting information). We see a gradual increase in the Mn^II^ signal, which results from accumulation of radiation-induced damage to the sample (see Fig. 4 ▸, and Fig. S2 of the supporting information). This is consistent with enhanced diffusion of radicals and secondary electrons at room temperature and thus increased XISD (O’Neill *et al.*, 2002 ▸; Kubin, Kern, Guo *et al.*, 2018 ▸). A similar scenario resulting in XISD with increasing X-ray dose was reported with Mn^III^(acac)_3_ in non-flowing solution at room temperature (Kubin, Kern, Guo *et al.*, 2018 ▸).

In the present study with the low flux setting at 30 K, we qualitatively estimate the radiation dose to be ∼88 kGy after the first scan (or pass), while for the spectra recorded with high flux setting this value is around 1.18 MGy. After three consecutive scans at the same spot, the total accumulated dose is 264 kGy (0.26 MGy) with the low flux setting (Fig. S3 of the supporting information). We further note that during the first scan with the low flux setting at a representative spot, when the monochromator moves to the Mn^II^*L*_3_-edge energy range, the Mn^III^(acac)_3_ sample already accumulates a dose of ∼30 kGy. This is one of the reasons for the presence of the Mn^II^ signal right from the first scan resulting from Mn^III^ → Mn^II^ photoreduction. Our estimated dose value with the low flux setting is roughly one order of magnitude higher than the reported dose value of 5 kGy for the damage-free PFY-XAS spectrum of Mn^III^(acac)_3_ (Kubin, Guo, Kroll *et al.*, 2018 ▸; Kubin, Kern, Guo *et al.*, 2018 ▸). Therefore, we would still need to reduce the radiation to minimize the photoreduction of Mn^III^ metal centers when employing our current raster scan approach at cryogenic temperature. We note that previous studies with liquid jet sample delivery methods that led to the collection of a damage-free Mn^III^ center require higher sample consumption. Therefore, it is difficult to probe precious metalloproteins, such as PS II, with this approach because of limited sample availability.

Although with our present approach we can limit the sample consumption, another issue arises relating to metal concentration. Typically, the metal concentration is significantly lower in these metalloproteins (in the range of sub m*M*) compared with the current model complex where the metal concentration is approximately 0.5 *M* (Fackler & Avdeef, 1974 ▸; Geremia & Demitri, 2005 ▸). Consequently, a reasonable signal would require a substantial amount of data acquisition time in the case of metalloproteins (about a week) with the present approach. However, increasing the sample area and a detector with ten times larger data collection area of our present detector would bring the data acquisition time down to a day.

The dose limits and the experimental parameters such as sample temperature and photon flux reported here can be used to assess other experimental approaches to reduce XISD. Previous XAS studies already reported a dose limit around 30 kGy to measure a nearly damage-free sample (Kubin, Guo, Kroll *et al.*, 2018 ▸; Kubin, Kern, Guo *et al.*, 2018 ▸). In the case of solid-state samples this damage could be lowered further by reducing the data acquisition time on each spot through optimizing the raster-based scan speed (Van Kuiken *et al.*, 2016 ▸; Van Kuiken *et al.*, 2018 ▸). Such controlled experiments with limited dose will be beneficial to studying metalloproteins and other higher-valence complexes.

Finally, we succeeded in measuring the *L*-edge RIXS map of Mn^III^(acac)_3_ (Fig. S4 of the supporting information). Further details of the RIXS setup are described elsewhere (Lee *et al.*, 2019 ▸). Such RIXS maps are element specific as these involve core transitions and provide unprecedented chemical details of the investigated molecular systems (Lundberg & Wernet, 2019 ▸; Kunnus *et al.*, 2016 ▸). Although the valence-excited final states, resulting from *L*-edge RIXS, are similar, to a first approximation, to those from UV–Vis spectroscopy, the ligand field transitions are often forbidden particularly for centrosymmetric complexes in the UV–Vis range. This is, however, not the case in *L*-edge RIXS, which shows intense metal-centered ligand field transitions. In addition, it is possible to clearly distinguish between charge transfer transitions (ligand-to-metal and metal-to-ligand) together with different intermediate and final spin-state information of the actual ligand field transitions from the RIXS map. Such detailed information is, however, not visible in our present RIXS data. One possible reason is the lower detector spectral resolution (∼1.5 eV), which, however, can be improved to 200 meV and even less in the future. Another reason could be the contribution resulting from radiation damage that leads to additional broadening.

## 4. Concluding remarks

In summary, in this work we report the Mn *L*-edge PFY-XAS spectra of solid-state Mn^III^(acac)_3_ at low temperatures of 30 K and 80 K, along with the room temperature spectra. At low temperatures, we immediately notice the Mn^II^ signal which results from X-ray induced damage. However, the contribution of the damage does not increase with increasing number of scans at the same spot, which we cannot fully explain at present. We speculate two possible reasons behind this phenomenon: (1) reduced diffusion of secondary radicals and subsequent recombination at low temperatures; (2) minimized diffusion of the chain propagation reaction. We note that our results are partially inconsistent because of the lack of a fully complete data set which is difficult to acquire as, in particular, it is difficult to vary numerous parameters systematically given the limited available beam time. In addition, our understanding of the underlying mechanisms is still incomplete. We see a slight increase in X-ray induced damage by increasing the temperature from 30 K to 80 K. At room temperature, we see a gradual increase in damage signal with increasing number of scans (passes) due to the accumulation of radiation dose. The primary goal of this work is to understand the viability of our present PFY-XAS approach using the TES detector to study the catalytic processes involving high-valent metal centers in various metalloenzymes such as PS II. Our current findings demonstrated that, although the TES detector successfully resolved the radiation-induced photoreduction of the high-valent Mn^III^ center, unfortunately the currently available implementation is not suitable for recording the PFY-XAS spectra of the high-valent Mn centers of the PS II sample. Using the dose limit and other experimental parameters discussed here, we validate other experimental approaches, such as, fs pulses from XFELs, which provide faster accumulation of signal compared with diffusion-driven damage and fast-flowing liquid jets at synchrotron sources. In addition, increasing the sample surface area and reducing dwell time on each spot will substantially reduce the radiation damage at synchrotron X-ray sources. These approaches may enable measurements of damage-free soft X-ray spectra of models and actual protein complexes, such as PS II. Finally, with the new LCLS-II facilities with improved pulse repetition rates of the order of tens of kHz leading to more efficient and faster data acquisition, we plan to apply *L*-edge RIXS to PS II. Such studies will allow the evolution of the spin-states of the metallic reaction center during the oxygen evolution process to be unraveled with unprecedented detail, which is beneficial for developments in artificial photosynthesis.

## Supplementary Material

Section S1 featuring Figurs S1 to S4. DOI: 10.1107/S1600577524012189/rv5186sup1.pdf
