# Supplementary material for: Probing soft X-ray induced photoreduction of a model Mn-complex at cryogenic conditions
Source: J Synchrotron Radiat. 2025 Feb 3;32(Pt 2):399–407. doi: 10.1107/S1600577524012189 (PMC11892889; doi:10.1107/S1600577524012189)
Supplement: Supplementary file 1 [file s-32-00399-sup1.pdf]

JOURNAL OF SYNCHROTRON RADIATION

Volume 32 (2025)

Supporting information for article:

Probing Soft X-ray Induced Photoreduction of a Model Mn-Complex at Cryogenic Conditions

Kuntal Chatterjee, Sang-Jun Lee, Li-Cheng Kao, Margaret D. Doyle, Charles J. Titus, Stephen R. Leone, Junko Yano, Vittal K. Yachandra, Philippe Wernet and Jan F. Kern

S1. Spectral fitting

We employ a least squares fit of a linear combination of intact PFY-XAS spectra of $Mn^{III}(acac)_3$ and $Mn^{II}(acac)_2$ published in Kubin *et al.* Chem. Sci., 2018, 9, 6813–6829. We normalize our spectra by the incident beam flux I0, consistent with the reported PFY. The damage-free spectra are broadened with a Gaussian convolution function with a full width half maximum (FWHM) of 0.59 eV to simulate the additional experimental broadening in our data. The incident energy bandwidth is estimated between 0.38-0.4 eV. The additional broadening is tentatively attributed to the other, probably minor, species present in the damaged sample. This causes our spectral features to appear slightly broader compared to the reported PFY-XAS spectra. This is why the sum contribution of these two species in the damaged spectrum is less than 100 % (our best fit gives this value as 93 %). We also note that the sample purchased is indicated to be 97 % pure.

The residuals obtained from our fitting as shown in Figure 2 in the manuscript are below 10 % for the spectra collected at 30 K and 80 K. While for the spectra collected at room temperature, the residuals are below 15 % except for the mismatch at peak positions of intact Mn^{II} and Mn^{III}, 639.6 eV and 641.6 eV, respectively.

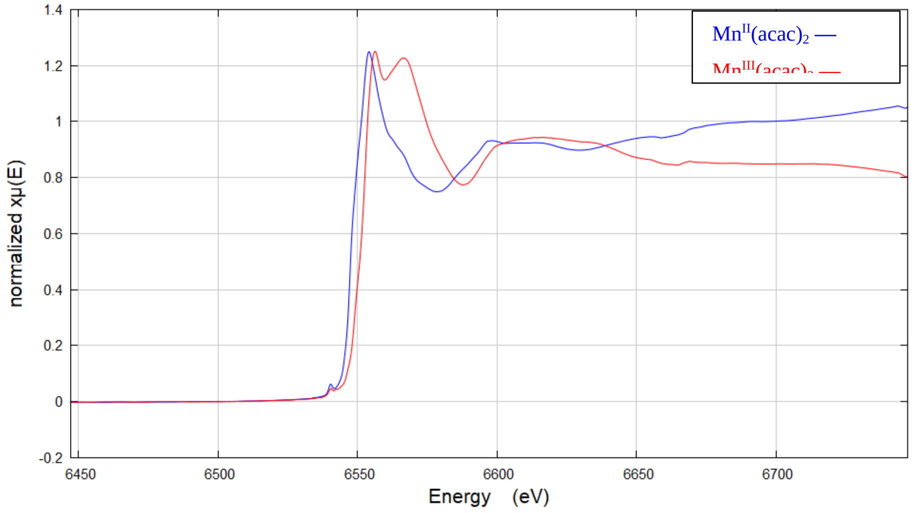

Figure S1 XANES spectra of $Mn^{III}(acac)_3$ and $Mn^{II}(acac)_2$ measured at Mn K-edge in transmission mode at SSRL beamline 7-3 at cryotemperature (~8 K). Two consecutive scans of each samples were measured at the same sample spot to make sure that there is no radiation damage.

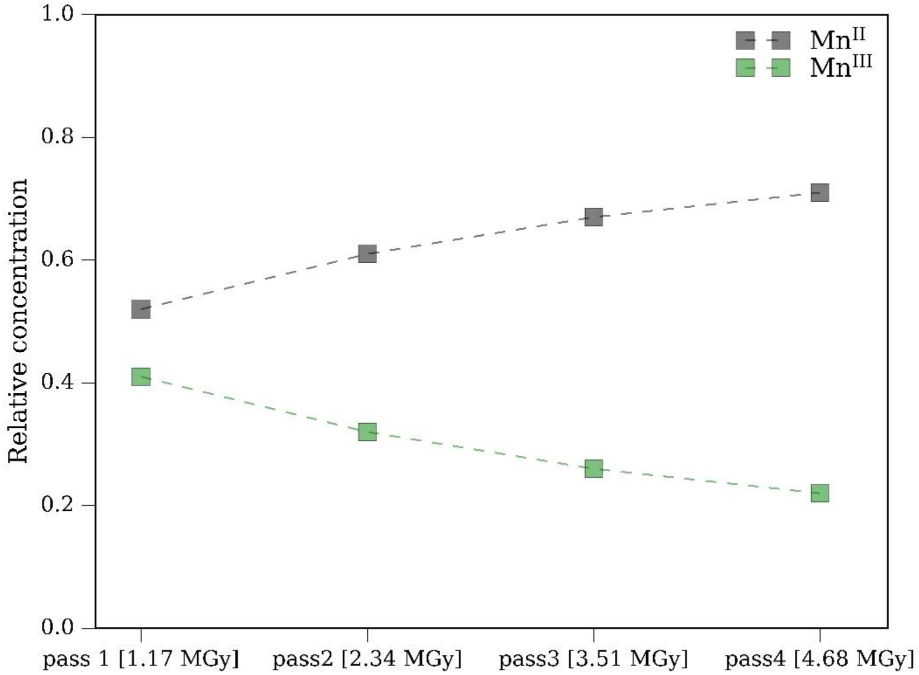

Figure S2 Gradual change in relative concentration of $Mn^{III}(acac)_3$ and $Mn^{II}(acac)_2$ with increasing number of scans at the same spot. The spectra are measured at room temperature. These spectra are collected with high flux setting 5 x 10^{10} photons/s. The accumulated dose after each pass/scan is mentioned as well.

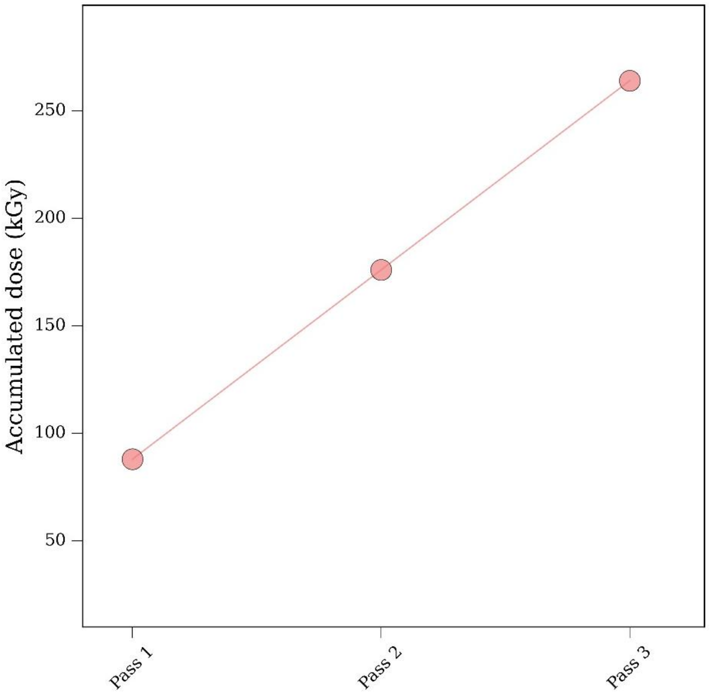

Figure S3 Dose accumulation at a spot with increasing number of pass/scans at 30 K with low flux settings and slit size 30 µm.

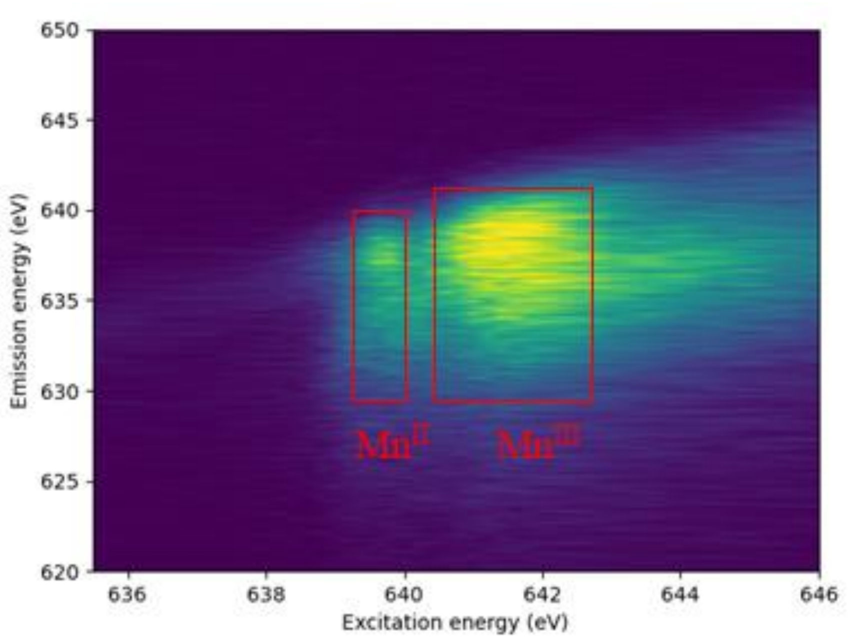

Figure S4 L-edge 2D resonant inelastic x-ray scattering (RIXS) map of the $Mn^{III}(acac)_3$. The emission energy is plotted as a function of the incident energy. We can see the contribution of both Mn^{II} and Mn^{III} in this map.
